# Subjective and Oxytocinergic Responses to Mindfulness Are Associated With Subjective and Oxytocinergic Responses to Sexual Arousal

**DOI:** 10.3389/fpsyg.2019.01101

**Published:** 2019-05-22

**Authors:** Janna A. Dickenson, Jenna Alley, Lisa M. Diamond

**Affiliations:** ^1^Program in Human Sexuality, Department of Family Medicine and Community Health, University of Minnesota, Minneapolis, MN, United States; ^2^Diamond Laboratory, Department of Psychology, University of Utah, Salt Lake City, UT, United States

**Keywords:** oxytocin, sexual arousal, mindfulness, attentional shifts, women, sexual health

## Abstract

Mindfulness – the ability to pay attention, on purpose, without judgment, and in the present moment – has consistently been shown to enhance women’s sexual arousal. As a first step toward understanding potential neuroendocrine underpinnings of mindfulness and sexual arousal, we examined whether individual differences in subjective and neuroendocrine (i.e., oxytocin) responses to mindful breathing were associated with individual differences in subjective and neuroendocrine responses to sexual arousal. To achieve this aim, 61 lesbian, bisexual, and heterosexual women completed a questionnaire assessing dispositional mindfulness, underwent an arousal task while continuously rating their sexual arousal and a mindful breathing task, after which participants reported on their ability to detect attentional shifts, and provided salivary samples after each assessment. Results indicated that women who were quicker to detect attentional shifts and women who reported greater sexual arousability reported larger changes (decreases) in oxytocin in response to mindful breathing and were the only women to report increases in oxytocin in response to the sexual arousal induction. Results further indicated that individuals who report greater subjective responsiveness to mindfulness and sexual arousal appear to have an oxytocinergic system that is also more responsive to *both* arousal and to mindfulness. These results make a significant contribution to our understanding of the role of attentional processes in sexual arousal, and warrant future examination of oxytocin as a potential neuroendocrine mechanism underlying the link between mindfulness and sexual arousal.

## Introduction

Female sexual arousal depends on one’s ability to attend to physical sensations and emotional experiences (Janssen et al., 2000; Spiering and Everaerd, 2007). Robust research has demonstrated that a specific form of attention – mindfulness – enhances sexual arousal. However, not all people are equally sensitive to mindfulness nor are all women equally *arousable* (i.e., the degree and ease in which women subjectively and nuerobiologically experience sexual arousal in response to sexual contexts). Yet, we have limited understanding of how such individual differences in arousability may be related to individual differences in *responsiveness to mindfulness*, involving the degree to which women subjectively respond (e.g., ease of attention, greater relaxation response) and the degree of neuroendocrine release in response to mindfulness inductions. One possibility is that those who are more responsive to mindfulness are also more arousable due to an increased biological sensitivity to contextual influences, marked by a neuroendocrine system with greater potential to benefit from mindfulness during mindful breathing and for greater arousability within a sexual context. Because oxytocin is involved in sexual arousal, enhances salience of social stimuli, and buffers the stress response, the oxytocinergic system may provide insight into why, how, or for whom mindfulness impacts arousal. The current study investigates whether lesbian, bisexual, and heterosexual women who are more responsive to mindfulness are also more arousable (responsiveness of the arousal system) and show larger changes in oxytocin to both a sexual arousal induction and a mindful attention induction.

Mindfulness – “to pay attention in a particular way: on purpose, in the present moment, and non-judgmentally” (Kabat-Zinn, 1990) – has received extensive investigation for its powerful stress-buffering effects across various mental health concerns (Grossman et al., 2004; Hofmann et al., 2010; Britton et al., 2012) and improving physical health outcomes (Kabat-Zinn et al., 1985; Davidson et al., 2003; Ludwig and Kabat-Zinn, 2008). Mindfulness involves intentionally bringing attention to one’s present moment thoughts, emotions, and sensations, with a spirit of curiosity, gentleness, and kindness. Training of mindfulness often begins with learning a mindful breathing exercise, in which practitioners gently focus their attention to the moment-to-moment physical sensations of breathing, allowing whatever physical sensations to be there, without judgment or attempting to control or change their experience in any way. Practitioners are taught that whenever their mind wanders away from their breath presents an opportunity to become mindful (Stahl and Goldstein, 2010). Training individuals to detect these attentional shifts (Doll et al., 2015) enhances attentional control (Dickenson et al., 2012; Hasenkamp et al., 2012; Kerr et al., 2013; Malinowski, 2013). By attending to, recognize, and label their present moment emotions, thoughts, and sensations, mindfulness practice also cultivates a compassionate relationship to one’s experience and promotes self-acceptance (Hollis-Walker and Colosimo, 2011; Keng et al., 2012).

Previous research has consistently linked trait mindfulness with various aspects of sexuality (Brotto et al., 2008; Adam et al., 2015a; Khaddouma et al., 2015; Arora and Brotto, 2017; Greer, 2017). Women with higher overall levels of trait mindfulness report greater sexual arousability (Mayland, 2005) and specific facets of trait mindfulness have been associated with various indices of sexuality. Women who report a greater tendency toward observing their internal thoughts, emotions, and sensations, notice their internal experiences without judgment (not labeling them as good or bad), and remain in present moment awareness (rather than behaving automatically) report greater sexual and relationship satisfaction (Khaddouma et al., 2015) and less sexual distress (Adam et al., 2015b). Additionally, present moment awareness, noticing internal experiences without judgment, and describing internal experiences with words reduce cognitive distractions that interfere with arousal and predict greater sexual functioning (Dunkley et al., 2015). These studies suggest that reporting greater tendencies to observe one’s experience in the present moment, without judgment contribute to greater overall levels of sexual arousal.

Mindfulness training also enhances arousal among women with various levels of arousability. For example, training mindful attention has proven effective in enhancing interoceptive awareness to sexual sensations and subjective sexual arousal among healthy women (Silverstein et al., 2011) and among women with sexual interest and arousal disorders (Brotto et al., 2016). Moreover, research has demonstrated that mindfulness training can have different impacts on different people (Arora and Brotto, 2017), suggesting that individual differences in women’s *responsiveness* to mindfulness practice may correspond to individual differences in women’s responsiveness to sexual contexts, or arousability.

Mindfulness may augment arousal via enhancing the salience of interoceptive stimuli in response to external sexual cues. For example, mindfulness training appears to have little effect on increasing physiological arousal, but increases the ability to attend to whatever physical sexual sensations are present, heightening the salience of interoceptive cues resulting in greater subjective arousal (Brotto et al., 2016). Additionally, improvements in sexual functioning resulting from mindfulness-based interventions are mediated by changes in trait mindfulness and overall levels of self-compassion (Paterson et al., 2017). The abovementioned research indicates that trait mindfulness is associated with greater sexual arousal, mindfulness training augments sexual arousability, and trait mindfulness is associated with greater responsiveness to mindfulness training. Such research indicates that the arousal-enhancing effects of mindfulness might stem from engendering a non-judgmental approach to observing one’s present moment thoughts, emotions, and sensations in response to internal and external cues. To date, a dearth of research has examined potential biological pathways that explain the links between trait mindfulness, responsiveness to mindfulness inductions, and sexual arousability. Oxytocin provides an ideal avenue for investigating links between individual differences in mindfulness (trait mindfulness and responsiveness to mindfulness practice) and individual differences in arousability, given its role in sexual behavior, stress reduction, and enhancing the salience of internal and external social stimuli.

Oxytocin is best known for the role that it plays in romantic and parental bonding (Carter, 1992). However, oxytocin also increases during sexual arousal and peaks during orgasm (Carmichael et al., 1987; Blaicher et al., 1999). Additionally, oxytocin is associated with greater subjective sexual arousal (Burri et al., 2008), contributes to the rewarding nature of sexual interactions (Veening et al., 2015), and integrates social attention, emotional feelings, and the functions of the autonomic system. Within its social function, oxytocin increases the salience of external social cues and interoceptive responses to social cues. Specifically, oxytocin affects the evaluation of emotions during early stages of information processing (Parr et al., 2013), modulates attentional orienting to social cues (Leknes et al., 2012), and buffers the stress response to social challenges (Heinrichs et al., 2003) by regulating cortisol responses (Devries et al., 2003), which allows individuals to approach challenges rather than avoid threats (Harari-Dahan and Bernstein, 2014). Moreover, oxytocin been linked to achieving greater emotional benefits from socially oriented forms of meditation practices, such as loving kindness (Isgett et al., 2016; Van Cappellen et al., 2016). Although the non-social effects of oxytocin has received much less attention, research has demonstrated that oxytocin facilitates the salience of personally relevant and emotional stimuli in non-social contexts (Harari-Dahan and Bernstein, 2014). For example, oxytocin facilitates a specific form of present-moment awareness – the feeling of becoming fully immersed in one’s experience (Piper et al., 2015; Pohling and Diessner, 2016). Because oxytocin facilitates salience of personally relevant and emotional stimuli, perhaps oxytocin facilitates non-judgmental approach to observing one’s present moment thoughts, emotions, and sensations in response to sexual cues and in response to mindfulness practice.

### Current Study

The aim of the current study was to examine whether oxytocin represents a potential biological pathway that elucidates the links between individual differences in trait mindfulness, responsiveness to mindfulness practice, and sexual arousability. To achieve this aim, 61 lesbian, bisexual, and heterosexual women reported their levels of trait mindfulness, completed a sexual arousal induction followed by a mindful breathing induction, and provided salivary oxytocin and cortisol samples. We used multilevel random coefficient modeling to examine the rate of change in oxytocin (measured after a baseline period, following the arousal and mindful breathing tasks, and after an unstructured recovery period) to answer the following objectives:

(1) *Does oxytocin change in response to a sexual arousal induction and a mindful breathing induction?* We predicted that oxytocin would increase in response to the sexual arousal and mindful breathing induction, relative to baseline.
(2) *Do the specific facets of trait mindfulness modulate the change in oxytocin in response to a sexual arousal induction and a mindful breathing induction?* We predicted that women who report a greater tendency to observe one’s thoughts, emotions, and sensations, notice their internal experiences without judgment, and remain in present moment awareness would show larger changes in oxytocin in response to the sexual arousal and mindful breathing inductions, relative to baseline.
(3) *Do subjective responsiveness to the sexual arousal and mindfulness inductions modulate change in oxytocin and do women who are more subjectively responsive have an oxytocinergic system that is broadly more responsive to various contexts* (i.e., *greater change in oxytocin irrespective of the type of induction*)*?* We predicted that women who report greater subjective responsiveness to the sexual arousal and mindfulness breathing inductions (i.e., greater arousability and quicker ability to detect attentional shifts) would report a more responsive oxytocinergic system irrespective of the type of induction, such that they would report larger changes in oxytocin in response to *both* the sexual arousal and the mindful breathing inductions, relative to baseline.
(4) *Are women who are subjectively and biologically more responsive to a mindfulness induction are also subjectively and biologically more sexually arousable?* We predicted that women who were more subjectively responsive to a mindful breathing induction would also report greater sexual arousability during a sexual arousal induction. We predicted that women who show larger changes in oxytocin in response to the sexual arousal induction also show larger changes in oxytocin in response to the mindful breathing induction.

## Materials and Methods

### Participants

Participants included women between the ages of 20 and 35 (mean age = 27.2). In all, 35% of the women identified as heterosexual, 44% as bisexual, and 21% as lesbian. In all, 91.1% of the women were White, 12.2% identified as Latina, 4.4% identified as Asian/Pacific Islander, 2.2% identified as Other Race, and 1.1% identified as African American. Participants were recruited through Facebook ads that described the study as an investigation of sexuality and stress hormones, and was approved by the University of Utah Institutional Review Board. Because two participants had incomplete data, they were dropped from analysis, resulting in a total of 61 participants. All participants had normal, natural menstrual cycle and were not taking any medications that could affected their sexual functioning (e.g., antidepressants). To control for digestive process on neuroendocrine release and time of day, participants were instructed to abstain from any food or caffeine intake approximately 2 h before their visit, which occurred between 3 pm and 7 pm.

### Procedures

Approximately 10 days after participants’ began menstruating, participants arrived at the laboratory to provide written informed consent and answer questions pertaining to their sexual history, current sexual attractions and experiences, and demographics. Women were then escorted to a private room to participate in the arousal induction task, where they underwent a 15 min baseline period. During the first 5 min, they sat quietly. During the second 5 min, they rated their liking of a set of landscape photographs, in order to engage their attention in a restful pleasant task (Jennings et al., 1992). During the remainder of the baseline period, they paced their breathing slowly in response to a timer (4 s of inhalation, 4 s of exhalation). Women then provided oxytocin and cortisol salivary samples.

Next, women listened to a series of neutral and erotic stories, which have been shown to reliably elicit sexual arousal among women (Chivers and Timmers, 2012). The stories varied by the nature of the interaction as well as the gender of the person in the story described. For example, each participant heard various stories that were depicted interacting with a man or a woman in sexual or non-sexual way (Chivers and Timmers, 2012). Each story was read to the participant through headphones, lasted about a minute and a half, and contained explicit language and situations. There was a one-min recovery period between each story, so that the total amount of time listening to the stories was 18 min. Throughout the duration of this task, women provided continuous ratings of their sexual arousal. Following this task, women provided a second salivary oxytocin sample, marking the index of oxytocin responsivity to sexual arousal.

Next, women underwent a focused breathing induction, in which participants were instructed to mindfully attend to their breath using a script adapted from previous research (Arch and Craske, 2006; Dickenson et al., 2012). Participants were instructed to attend to the moment-by-moment physical sensations of their breath and whenever their mind wanders to congratulate yourself each time on reconnecting with your experience in the moment and gently escorting the attention back to the breath. Following this task, women completed a series of questions about their experience during the mindful breathing task and provided the third salivary sample, which marks oxytocin responsivity to mindful breathing. They were then escorted back to the open room where spent an additional 15 min sitting quietly and filling out additional questionnaires and were oriented to additional aspects of the study. Participants provided their final salivary sample, which marks the “recovery” period. Hence, each of the four saliva samples were collected 15–20 min apart.

Participants additionally completed a secondary laboratory assessment that was nearly identical to the first session with one exception. After the baseline period, women underwent a modified version of the Trier Social Stress Test (Kirschbaum et al., 1993), including the performance of a speech and the completion of a difficult serial subtraction task in the presence of an experimenter. This task is not discussed in the current study, other than serving to compare oxytocin responses during mindful breathing to explore potential order effects. After this session, participants were debriefed and reimbursed for their time. This study was approved by University of Utah Institutional Review Board.

### Task Based Measures

#### Subjective Arousability

Participants recorded their ratings for arousal continuously throughout the arousal task. In order to obtain a measure of *responsiveness* to the arousal induction (or arousability), we used peak ratings from the erotic stories. Given that we included lesbian, bisexual, and heterosexual women, we included erotic stories that depicted men and stories that depicted women. We computed women’s self-reported peak ratings for each of the erotic stories featuring their self-reported preferred gender (inferred from self-report of their attractions) and for each of the erotic stories featuring their non-preferred gender.

#### Subjective Responsiveness to Mindful Breathing

Following the mindful breathing task, participants rated their extent of agreement with a series of statements including “I was restless and bored during this experience,” “I felt calm and relaxed,” and “It took me a while to notice that my mind had wandered.” Because the former questions were highly correlated, we aggregated these scores into a composite measure of relaxation response to mindful breathing. Given that being able to detect when one’s mind has wandered is a central component of mindfulness training, we examined the mind wandering question as a single-item measure indicative of one’s ability to detect attentional shifts. This item was reverse coded such that higher scores indicated being quicker to detect attentional shifts.

#### Trait Mindfulness

To limit participant burden of completing myriad questionnaires, we used a modified version of the five facet mindfulness questionnaire (FFMQ), which assessed 25-items of the FFMQ, corresponding to five related dimensions of trait mindfulness. All items of the FFMQ are rated on a 5-point Likert scale, ranging from 1 (*never or very rarely true*) to 5 (*very often or always true*). All subscales corresponding to the dimensions had good reliability. The observing subscale (α = 0.78) indicates the degree of observing (attending or noticing) internal (e.g., bodily sensations, thoughts, and emotions) and external sensations (e.g., smells, sights, and sounds). The Describing subscale (α = 0.84) reflects the ability to express in words one’s own experience. The acting with awareness (α = 0.85) subscale reflects attending to one’s present moment activity, rather than behaving automatically, without much thought or attention and is the subscale that mirrors constructs closest to other trait mindfulness scales (e.g., MAAS; Carlson and Brown, 2005). The non-judging of Inner Experience (α = 0.89) subscale reflects an ability to accept thoughts and emotions without judging them (i.e., evaluating as good or bad). The non-reactivity to Inner Experience (α = 0.77) subscale reflects the ability to detach from one’s inner experience (i.e., thoughts and emotions) and allowing thoughts and emotions to come and go without clinging to or avoiding them. Because these five facets are related, but distinct constructs, separate scores for each subscale were calculated to be entered into the analytic models (non-significant subscale scores were removed from statistical models).

#### Endocrine Measures

For the collection of salivary oxytocin data, participants collected approximately 1 mL of saliva in their mouths and released the saliva into a glass centrifuge tube using the “passive drool” technique. Samples were immediately frozen at −25°C until shipped on dry ice to the University of North Carolina, Chapel Hill. The oxytocin enzyme-immunoabsorbance (EIA) method used to assay salivary oxytocin is identical to that reported previously (Holt-Lunstad et al., 2008). Salivary oxytocin levels were measured using the oxytocin EIA (Enzo Life Sciences, Farmingdale, NY, United States). After correcting for concentration produced by extraction, the lower limit of sensitivity was 1.5 pg/mL, with intra- and inter-assay variations of 4.8 and 8%.

The current study also controlled for cortisol levels, given that oxytocin has been linked to cortisol. The specific link between cortisol and oxytocin are reported elsewhere (Alley et al., 2019) and, thus, were not the focus of the current study. Salivary cortisol samples were taken using salivettes (Sarstedt, Germany), consisting of a plastic tube with a cotton insert. The participant was instructed to lightly chew on the insert to thoroughly soak it with their saliva. All samples were kept frozen at −25°C until being shipped on dry ice to be assayed by the laboratory of Dr. Kirschbaum at the Technical University of Dresden, which uses a time-resolved immunoassay with fluorometric end point detection (see Dressendorfer et al., 1992) with intra- and inter-assay precision of 3.0 and 4.2%. In all, 7% of cortisol samples were either missing or could not be assayed, and follow-up analyses detected no systematic patterns of missing samples (i.e., no correlations with other study variables).

### Analytic Strategy

Analyses were conducted with multilevel random coefficient modeling (MRCM, employed with WHLM; Bryk and Raudenbusch, 1992), to represent the nested nature of the data, in which lower level units (oxytocin) vary within persons. Specifically, piecewise linear growth modeling was conducted to examine changes in oxytocin levels across each time period, which allows for the division of growth trajectories into separate linear components and is a common approach to examine responsivity and recovery processes. The first linear component (responsivity), coded 0 as baseline and 1 for the remaining 3 time points, represents the change between baseline, and arousal/stress responses. The second linear component, coded 0 for baseline and for the arousal/stress inductions and 1 for the remaining tasks, represents the rate of change in oxytocin during the mindful breathing induction. The third linear component, coded 0 for the first three samples and 1 for the recovery sample, represents the rate of change in oxytocin at the end of the visit (recovery). Because of positive skew, both oxytocin and cortisol were log-transformed for analysis. The Level 1 model has the following structure:

Oxytocin_*timet,participanti*_ = π_0_*_i_* + π_1*i*_(Sexual Arousal) + π_2_*_i_*(Mindful Breathing) + π_3_*_i_*(Recovery) + π_4_*_i_*(Cortisol) + *e_ti_*

where π_0_*_i_* represents baseline oxytocin, π_1_*_i_*, π_2_*_i_*, and π_3_*_i_* represent the rate of linear change in oxytocin across the sexual arousal induction, rate of change during mindful breathing, and rate of change following a recovery period, and *e_ti_* represents the within-individual error in participant *i*’s oxytocin that cannot be accounted for by cortisol levels (π_4_*_i_*), baseline oxytocin (π_0_*_i_*), or by change over time (π_1_*_i_*, π_2_*_i_*, and π_3_*_i_*).

These Level 1 associations were then modeled as a function of an intercept and a random effect at Level 2 to obtain overall estimates of baseline and rate of change in oxytocin, controlling for cortisol levels. Non-significant random effects were dropped from this unconditional model. Next, we modeled the Level 1 associations as a function of Level 2 variables to examine whether the size and/or direction of these effects varies from woman to woman.

The Level 2 model calculated whether women who showed greater subjective sexual arousal, greater subjective responses to mindful breathing, and higher levels of trait mindfulness showed differences in their oxytocin levels at baseline, in response to the sexual arousal induction, and in response to mindful breathing. Level 2 variables were grand centered, such that the Level 2 intercepts represent the average rate of change in oxytocin at average (sample mean) levels of peak sexual arousal, subjective responses to mindful breathing, and trait mindfulness. To achieve the most parsimonious model to test our hypotheses, oxytocin recovery and the link between oxytocin and cortisol were modeled as a function of the intercept and error. As well, variables (representing level 2 moderators of level 1 associations) that were non-significant were dropped from the model, resulting in the final level 2 model that predicted rate of change in oxytocin from trait mindfulness (present moment awareness and noticing internal experiences without judgment), detecting attentional shifts, and sexual arousability toward the preferred sex. Significant Level 2 effects were followed up with simple slopes at 25th and 75th percentiles around the mean of each continuous moderator. Additionally, because previous research has found that the facets of trait mindfulness may interact to predict outcomes (Eisenlohr-Moul et al., 2012), we also examined interactions between non-reactivity and non-judgment with observing thoughts, emotions, and sensations and present moment awareness. However, these results were insignificant and were dropped from our analytic model.

## Results

Table 1 presents means, standard deviations, and bivariate correlations among the primary study variables. Bivariate correlations indicated that women who reported greater sexual arousability toward their preferred sex showed greater tendency to put their experiences into words (describing) and overall trait mindfulness (full scale FFMQ).

**Table 1:** Descriptive and correlations between study variables.

	Mean	*SD*	1	2	3	4	5	6	7	8	9	10	11	12	13	14	15	16	17	18
1 Peak arousal toward preferred sex	6.13	2.08	–																	
2 Peak arousal toward non-preferred sex	4.65	2.32	0.34^∗^	–																
3 Average of all peak arousal	4.86	1.72	0.77^∗^	0.81^∗^	–															
4 Observing	21.82	5.01	0.14	0.19	0.24	–														
5 Describing	13.2	3.38	0.28^∗^	0.26^∗^	0.29^∗^	0.08	–													
6 Act with awareness	19.31	3.45	0.20	0.14	0.18	−0.16	0.21	–												
7 Non-judging	15.66	3.50	0.14	−0.03	0.10	−0.18	0.11	0.33^∗^	–											
8 Non-reacting	12.64	3.30	0.03	−0.13	−0.06	0.19	−0.06	0.23	0.37^∗^	–										
9 Full scale FFMQ	3.30	0.39	0.28^∗^	0.18	0.31^∗^	0.48^∗^	0.48^∗^	0.54^∗^	0.54^∗^	0.62^∗^	–									
10 Mind wandering	3.74	0.96	0.04	0.19	0.13	0.02	0.05	0.16	−0.24	−0.22	−0.08	–								
11 Mindful relaxation	4.07	1.07	0.25^∗^	0.24	0.27^∗^	0.21	0.00	0.09	0.21	0.23	0.29^∗^	0.04	–							
12 Oxytocin – baseline	11.20	7.00	−0.19	0.00	−0.08	0.02	−0.01	−0.30^∗^	0.00	−0.07	−0.12	−0.27^∗^	−0.05	–						
13 Oxytocin – arousal	11.74	6.43	−0.10	−0.03	−0.08	−0.05	−0.01	−0.28^∗^	0.05	−0.12	−0.15	−0.20	0.03	0.85^∗^	–					
14 Oxytocin – mindfulness	10.53	6.51	−0.10	0.02	−0.04	0.02	−0.03	−0.16	0.03	−0.13	−0.09	−0.20	−0.04	0.84^∗^	0.89^∗^	–				
15 Oxytocin – recovery	11.73	6.18	−0.30^∗^	−0.12	−0.25	−0.10	0.00	−0.21	−0.08	−0.17	−0.21	−0.29^∗^	−0.15	0.80^∗^	0.82^∗^	0.78^∗^	–			
16 Cortisol – baseline	8.86	5.3	−0.02	−0.01	−0.08	−0.08	0.00	−0.04	−0.30^∗^	−0.14	−0.21	−0.21	−0.09	0.25	0.17	0.17	0.23	–		
17 Cortisol – arousal	6.61	5.11	−0.08	−0.13	−0.13	−0.03	−0.14	−0.08	−0.23	−0.09	−0.21	−0.08	−0.11	0.21	0.11	0.14	0.15	0.62^∗^	–	
18 Cortisol – mindfulness	6.38	4.64	−0.01	−0.02	−0.03	−0.11	−0.12	−0.02	−0.18	−0.16	−0.22	−0.05	−0.04	0.26^∗^	0.27^∗^	0.28^∗^	0.23	0.55^∗^	0.85^∗^	–
19 Cortisol – recovery	6.41	5.13	0.03	0.03	0.00	−0.14	0.03	−0.03	−0.23	−0.14	−0.20	−0.02	0.03	0.20	0.28^∗^	0.14	0.19	0.60^∗^	0.67^∗^	0.81^∗^

*^∗^p < 0.05.*

### Objective 1: Does Oxytocin Change in Response to the Sexual Arousal and Mindful Breathing Inductions? No and Yes

The unconditional model examined the rate of change in oxytocin across tasks. Contrary to our predictions, oxytocin did not significantly change in response to the sexual arousal induction (*p* > 0.1). However, there was a significant random effect [χ^2^(62, *N* = 63) = 178.38, *p* < 0.001], indicating that women varied in the extent to which oxytocin changed in response to the sexual arousal induction. In contrast to our objective that oxytocin would increase in response to mindful breathing, oxytocin significantly *decreased* in response to mindful breathing (*b* = −0.13, *SE* = 0.05, *p* = 0.011). We also found that change in oxytocin in response to mindful breathing varied significantly across women [χ^2^(62, *N* = 63) = 110.93, *p* < 0.001]. Following the mindfulness induction, during the recovery period, oxytocin increased, and surpassed baseline levels of oxytocin (*b* = 0.12, *SE* = 0.05, *p* = 0.01). The degree to which oxytocin increased during the recovery period varied significantly across women [χ^2^(62, *N* = 63) = 105.07, *p* < 0.001]. In summary, oxytocin did not significantly increase in response to the sexual arousal induction, decreased significantly in response to mindful breathing, and increased (passed baseline) during recovery.

#### Replication of Decreases in Oxytocin in Response to Mindful Breathing

Given the unexpected finding that mindful breathing *decreased* rather than increased oxytocin, we were concerned the nature of the arousal task may have influenced the direction of change in oxytocin in response to mindful breathing and whether this pattern was replicable. Thus, we examined the replicability of this pattern by examining the pattern of oxytocin in response to a laboratory stressor 2 weeks later. Similar to the first visit, oxytocin significantly decreased in response to the mindful breathing induction (*b* = −0.14, *SE* = 0.04, *p* = 0.002) and the rate of change varied significantly across women [χ^2^(63, *N* = 62) = 97.16, *p* = 0.003]. For those interested in the effects on stress, oxytocin increased in response to stress (*b* = 0.12, *SE* = 0.04, *p* = 0.002) and returned to baseline levels at recovery (*p* > 0.2). The visual pattern of oxytocin across the second visit (not shown) mirrored the pattern during the first visit; oxytocin increased during stress, decreased during mindful breathing, and returned to baseline during recovery.

**FIGURE 1:**
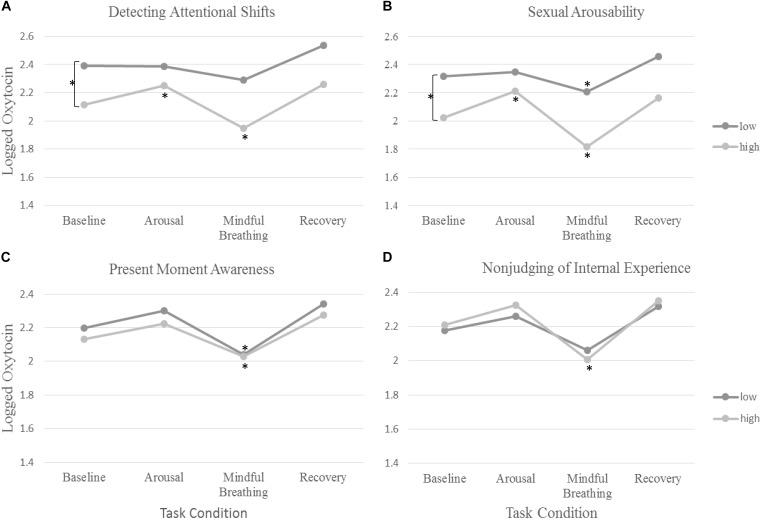
The rate of change in oxytocin across tasks and differences across **(A)** detecting attentional shifts, **(B)** subjective sexual arousability, **(C)**, present-moment awareness, and **(D)** non-judging of internal experience. The symbol Asterisks (^∗^) denote significance in change in oxytocin from baseline.

Given that women significantly differed in how oxytocin changed across tasks, we next examined whether the facets of trait mindfulness and subjective responsiveness to the mindful breathing and sexual arousal inductions moderated the responsiveness of oxytocin – the rate of change in oxytocin across tasks (see Figure 1 and Table 2). Given that sexual arousal has shown to differ across sexual orientation and relationship status (Lippa, 2006), we initially controlled for sexual orientation and relationship status. Consistent with standard practice, we dropped non-significant level 2 moderators (subjective sexual arousability for the non-preferred sex, observing thoughts, emotions, and sensation, describing internal experiences with words, non-reacting to internal experiences, relaxation responsiveness during mindful breathing, sexual orientation, and relationship status).

**Table 2:** Multilevel model assessing subjective responses as moderators of the rate of change in oxytocin.

Fixed effect	Coeffi cient	Standard error	*t*-ratio	*p*-value
**Baseline oxytocin, π_0_**				
Intercept, β_*00*_	2.23	0.10	21.703	<0.001
Act with awareness, β_*01*_	−0.03	0.03	−1.256	0.214
Non-judging of internal experience, β_*02*_	0.01	0.03	0.253	0.801
Detecting attentional shifts, β_*03*_	−0.28	0.09	−3.127	0.003
Peak sexual arousal, β_*04*_	−0.11	0.05	−2.251	0.028
**Rate of change in oxytocin to arousal, π_1_**				
Intercept, β_1*0*_	0.10	0.06	1.69	0.097
Act with awareness, β_*11*_	0.00	0.01	−0.37	0.712
Non-judging of internal experience, β_*12*_	0.01	0.01	0.665	0.509
Detecting attentional shifts, β_*13*_	0.14	0.06	2.371	0.021
Peak sexual arousal, β_*14*_	0.06	0.03	2.176	0.034
**Rate of change in oxytocin to mindful breathing, π_2_**				
Intercept, β_*20*_	−0.15	0.05	−3.188	0.002
Act with awareness, β_*21*_	0.03	0.01	3.432	0.001
Non-judging of internal experience, β_*22*_	−0.02	0.01	−2.651	0.01
Detecting attentional shifts, β_*23*_	−0.07	0.03	−2.137	0.037
Peak sexual arousal, β_*24*_	−0.04	0.02	−2.07	0.043
**Rate of change in oxytocin after recovery, π_3_**				
Intercept, β_*30*_	0.14	0.05	3.03	0.004
Cortisol, π_*4*_				
Intercept, β_*40*_	−0.03	0.02	−1.743	0.083

### Objective 2: Do Women With Greater Trait Mindfulness Show Larger Changes in Oxytocin in Response to Sexual Arousal and Mindful Breathing? No and Yes

None of the facets of trait mindfulness moderated the change in oxytocin in response to the sexual arousal induction (*p* > 0.2). However, we found that two facets of trait mindfulness (Non-judging of internal experiences and acting with awareness) moderated the rate of change in oxytocin in response to mindful breathing. As expected, women who reported greater levels of Non-judging of Internal Experiences showed larger changes (decreases) in oxytocin in response to mindful breathing (*b* = −0.02, *SE* = 0.01, *p* = 0.010; see Table 2). Simple slopes revealed that women who reported lower levels Non-judging of Internal Experiences showed the least change in oxytocin in response to mindful breathing (*p* > 0.06).

Women who reported greater levels of Acting with Awareness showed weaker decreases in oxytocin in response to mindful breathing (*b* = −0.07, *SE* = 0.03, *p* = 0.037; see Table 2). Graphical analysis (see Figure 1) and simple slopes revealed that women who reported higher levels of acting with awareness showed lower levels of oxytocin at baseline (*b_highAware_* = 2.24, *SE_highAware_* = 0.11; *b_LowAware_* = 2.17, *SE_LowAware_* = 0.12; χ^2^(2) = 411.61, *p* < 0.001) but similar levels of oxytocin in response to mindful breathing as women who reported lower levels of Acting with Awareness (*b_highAware_* = 2.07, *SE_highAware_* = 0.13; *b_LowAware_* = 2.08, *SE_LowAware_* = 0.12). Hence, the weaker (but still significant) decreases among women with higher levels of Acting with Awareness, relative to those with lower levels of Acting with Awareness may have been a function of differences in baseline levels. However, these baseline differences evidenced in simple slopes tests were not significant in full model, which controls for but does not include oxytocin responses to mindful breathing (see Table 2).

In summary, women with higher levels of Non-judging of Internal Experiences showed larger changes in oxytocin in response to mindful breathing. Those with lower levels of Acting with Awareness showed larger changes in oxytocin in response to mindful breathing, but this effect may have been due to baseline differences rather than absolute levels of oxytocin during mindful breathing. Moreover, trait mindfulness was not related to change in oxytocin to the sexual arousal induction.

### Objective 3A: Do Women Who Are Subjectively More Responsive to the Mindfulness Induction (or Sexual Arousal Induction) Show Larger Changes in Oxytocin in Response to the Mindfulness Induction (or Sexual Arousal Induction)? Yes

First, we examined whether women who are subjectively more responsive to the mindfulness induction show larger changes in oxytocin in response to the mindfulness induction. Women who felt more or less calm and relaxed during the mindful breathing induction did not show any differences in their rate of change in oxytocin. However, women who were quicker to detect attentional shifts during mindful breathing moderated the rate of change in oxytocin. We found that women who were quicker to detect attentional shifts during mindful breathing had lower levels of oxytocin at baseline (*b* = −0.28, *SE* = 0.09, *p* = 0.003; see Figure 1 and Table 2). Consistent with our hypotheses, we found that women who were quicker to detect attentional shifts during mindful breathing showed larger changes (decreases) in oxytocin in response to mindful breathing (*b* = −0.07, *SE* = 0.03, *p* = 0.037; see Figure 1 and Table 2). Simple slopes revealed that only women were slow to detect when their mind wandered showed no change in their oxytocin in response to mindful breathing (*p* > 0.06).

Then, we examined whether women who are subjectively more responsive to the sexual arousal induction show larger changes in oxytocin in response to the sexual arousal induction. We found that women who were more arousable toward their preferred sex showed lower levels of oxytocin at baseline (*b* = −0.11, *SE* = 0.05, *p* = 0.028; see Table 2). Consistent with our hypotheses, we found that women who were more arousable showed larger changes in oxytocin in response to the sexual arousal induction (*b* = 0.06, *SE* = 0.03, *p* = 0.034; see Table 2). Simple slopes indicated that those with low or average levels of sexual arousability showed no significant change in oxytocin (see Table 2), whereas those who showed high levels of sexual arousability showed significant increases in oxytocin in response to the sexual arousal induction (*b_HighArousability_* = 0.19, *SE_HighArousability_* = 0.07; χ^2^(1) = 7.07, *p* = 0.008). In summary, subjective responsiveness to mindfulness was associated with change in oxytocin in response to mindful breathing and subjective arousability was associated with change in oxytocin in response to the sexual arousal induction.

### Objective 3B: Is the Moderating Effect of Subjective Responsiveness on Change in Oxytocin Specific to the Type of Induction or Do Women Who Are More Subjectively Responsive Have an Oxytocinergic System That Is Broadly More Responsive to Various Contexts? the Latter

We examined whether women who are subjectively more responsive to the mindfulness induction show larger changes in oxytocin in response to the sexual arousal induction. Consistent with our hypothesis, women who were quicker to detect attentional shifts during mindful breathing showed larger changes in oxytocin during the sexual arousal induction (*b* = 0.14, *SE* = 0.06, *p* = 0.021; see Figure 1 and Table 2). Simple slopes indicated that those with low or average levels of detecting attentional shifts showed no significant change in oxytocin during the sexual arousal induction (see Table 2). However, women who were quicker to detect attentional shifts during mindful breathing showed significant increases in oxytocin in response to the sexual arousal induction (*b_HighMindful_* = 0.14, *SE_HighMindful_* = 0.06; χ^2^(1) = 5.75, *p* = 0.016).

Next, we examined whether women who are subjectively more responsive to the sexual arousal induction show larger changes in oxytocin in response to the mindfulness induction. Consistent with this objective, women who were more subjectively arousable showed larger changes (decreases) in oxytocin in response to mindful breathing (*b* = −0.04, *SE* = 0.02, *p* = 0.043; see Figure 1 and Table 2).

In summary, results of hypotheses 3A,B indicate that women who were more subjectively responsive to the sexual arousal induction and women who were more subjectively responsive to the mindful breathing induction showed larger changes in oxytocin, irrespective of the type of induction. Thus, we next examined whether women who are more responsive to the sexual arousal induction are also the same women who are more responsive to the mindfulness induction.

### Objective 4: Are Women Who Are Subjectively and Biologically More Responsive to Mindful Breathing Also Subjectively and Biologically More Responsive to Erotic Stimuli? No and Yes

To investigate whether women who subjectively report greater responsiveness to the mindfulness induction also report greater responsiveness to the sexual arousal induction, we investigated the bivariate correlations shown in Table 1. Women who were more arousable showed greater relaxation responses to the mindful breathing, but did not show any differences in their ability to detect attentional shifts (see Table 1).

We next assessed whether women who show larger changes in oxytocin in response to the sexual arousal induction also show larger changes in oxytocin in response to the mindful breathing induction. To obtain this correlation, we first outputted the residuals from the unconditional model, which represent the variation in the rate of change in oxytocin across individuals from the expected linear slope. We used linear regression to predict residuals of the rate of change in oxytocin to the mindful breathing induction from the residuals of the rate of change in oxytocin in response to the sexual arousal induction, controlling for residuals of oxytocin at baseline. In support of our hypothesis, change in oxytocin in response to the sexual arousal task, but not baseline oxytocin, predicted change in oxytocin in response to the mindful breathing induction (*b* = −0.25, *SE* = 0.09, *t*(60) = −2.95, *p* = 0.005). Specifically, women who showed larger increases in oxytocin in response to the sexual arousal induction showed larger decreases in oxytocin in response to mindful breathing.

In summary, women who were subjectively more arousable show greater relaxation responsiveness to mindful breathing, but relaxation responses to mindful breathing were not associated with change in oxytocin. In contrast, attentional responsiveness to mindful breathing (detecting attentional shifting) was associated with change in oxytocin to both the sexual arousal and mindful inductions, but was not associated with subjective arousability. Finally, women who showed greater changes in oxytocin in response to mindful breathing showed greater changes in oxytocin in response to the sexual arousal induction.

## Discussion

The current study sought to understand whether individual differences in responsiveness to mindful breathing were associated individual differences in responsiveness to a sexual arousal induction. We found that oxytocin significantly decreased during mindful breathing and increased during a recovery period, with no significant changes observed during the sexual arousal induction. Importantly, these main effects were heavily moderated. For example, women who were more attentive during mindful breathing and women who were more arousable during the sexual arousal induction were the only individuals who showed increases in oxytocin during the sexual arousal task. Such results make a significant contribution to our understanding of female sexual arousability by suggesting a potential role of oxytocin in linking mindfulness to sexual arousal.

We sought to understand whether some women reported a biological sensitivity to contextual influences, marked by a greater potential for mindfulness when engaging in mindful breathing and a greater potential for arousability during sexual contexts. On one hand, our results corroborate this objective by indicating that women who reported greater subjective arousability and subjective responsiveness to mindful breathing (detecting attentional shifts) reported larger changes in oxytocin in response to *both* mindful breathing and sexual arousal. This indicates that the moderating effects of subjective responsiveness on changes in oxytocin were not specific to the type of induction. Rather, women who subjectively respond more to mindful breathing and sexual arousal appear to have an oxytocinergic system that is generally more responsive (i.e., showed larger changes to both sexual and mindfulness contexts). Moreover, women who showed increases in oxytocin in response to the sexual arousal induction showed larger decreases in response to the mindful breathing induction. The culmination of these findings suggest that women who reported greater responsiveness to the arousal and mindful breathing inductions have an oxytocinergic system that is more responsive to *both* arousal and to mindfulness.

On the other hand, we found that the aspects of mindfulness that were associated with subjective arousability were not the same aspects of mindfulness that were associated with oxytocin responses to the sexual arousal induction. Specifically, women who reported greater subjective arousability showed greater relaxation responses during mindful breathing, but showed no differences in detecting attentional shifts, and the opposite was true for women with greater responsiveness in oxytocin. Thus, women who are more arousable may have an oxytocin system that is sensitive to both mindfulness and sexual arousal, but women who report greater arousability do not subjectively respond to the same aspects of mindfulness that are relevant to oxytocin. Relatedly, women with greater trait mindfulness (e.g., intentionally bringing present moment awareness to daily activities, observing without judging) showed larger changes in oxytocin in response to mindful breathing, but these facets were unrelated to subjective responses to the mindfulness induction and subjective and oxytocin responses to the sexual arousal induction. Although our results demonstrate links between oxytocin, mindfulness, and sexual arousability, the specific aspects of mindfulness practice relevant to oxytocin, trait mindfulness, and sexual arousability differ from one another. Perhaps such discrepancies represent important distinctions in the nuances of how mindfulness relates to subjective experiences and neuroendocrine release. However, we cannot rule out whether these results may have been due to reducing the number of items on the FFMQ or to the lack of assessing in-the-moment interoceptive awareness, which has been shown to mediate the link between mindfulness-based interventions for sexual interest and arousal disorders (see Arora and Brotto, 2017). Future research should use well-validated measures that assess various dimensions of mindfulness (Mehling et al., 2012; Gu et al., 2016) in order to examine the indirect pathways through which oxytocin impacts the link between various aspects of mindfulness (facets of trait mindfulness, attentional, and emotional responsiveness to mindfulness inductions) and sexual arousal.

Perhaps the most surprising finding from our study was that mindful breathing *decreased* oxytocin. Ancillary analysis indicated that this effect was replicated across two separate time points, indicating that oxytocinergic decreases due to mindful breathing were not task or time specific, but marked reliable biological indicators of change due to mindful breathing. Decreases in oxytocin resulting from mindful breathing were further corroborated by results indicating that individuals who were better able to detect attentional shifts during mindful breathing not only showed greater decreases in oxytocin during mindful breathing, but also showed lower levels of baseline oxytocin. A similar, though more complicated, pattern emerged with trait mindfulness: those who were less judgmental of their internal experiences showed greater decreases in oxytocin in response to mindful breathing and those who acted with greater present-moment awareness showed lower levels of oxytocin across tasks but less – albeit still significant – decreases in oxytocin in response to mindful breathing. Whereas most of the research on oxytocin focuses on *increases* in oxytocin, the current data indicate that *reductions* in oxytocin may also be important for some tasks (i.e., mindful breathing).

Given the dearth of research on mindfulness and oxytocin and the potential benefits of oxytocinergic decreases, the exact meaning of the abovementioned effect remains unclear. However, Keeler et al. (2015) found that oxytocin increases or decreases depending on the degree of social attunement and responsive communication required in the task. Such findings speak to a differential effect of oxytocin depending the specifics of the task. Given that oxytocin plays a role in sexual arousal, orgasm, pair-bonding (Borrow and Cameron, 2012; Veening et al., 2015), social affiliation, emotional elevation (Piper et al., 2015; Pohling and Diessner, 2016), and stress (Taylor, 2006; Taylor et al., 2006; Chen et al., 2011; Kumsta and Heinrichs, 2012; Olff et al., 2013; Seltzer et al., 2014), it is possible that increases in oxytocin are necessary for stimulating and activating an individual to meet to the demands for sexual and social interactions. This is consistent with the presumed role of oxytocin to help organize an organisms response to the demands of a stressor or social situation by stimulating arousal and motivate an organism to seek out help and assistance from others when needed (Taylor, 2006; Kumsta and Heinrichs, 2012; Olff et al., 2013; Seltzer et al., 2014).

If increases in oxytocin are uniquely beneficial when the task requires some degree of activation, stimulation, or arousal (i.e., activation of the sympathetic system), our findings indicate that mindful breathing does not require the activation that increases in oxytocin provides, but rather is associated with decreases in oxytocin. Although mindfulness can stimulate general arousal (Ditto et al., 2006), the arousal processes involved in mindfulness practice appears to be more related to learning and memory, rather than mobilizing the arousal response to action, as indicated by theta wave activity and slowing of alpha wave activity (see Ivanovski and Malhi, 2007 for a review). Mindful breathing tends to rely more on a regulatory system thought to be mediated by the vagus nerve (Gao et al., 2016), responsible for physiological recovery, digestion, and rest. Considering mindfulness inductions as an enhanced recovery phase may explain why mindful breathing decreased oxytocin. Recent research indicates that oxytocin during stress was associated with faster vagal recovery, suggesting that oxytocin may actually boost recovery to stress more than it buffers reactivity (Engert et al., 2016). Hence, a decrease in oxytocin during mindful breathing might represent that effective recovery processes have been achieved. Given that sexual arousal induction activates similar processes as stress (e.g., both activate arousal), this may also explain why women who showed larger increases in oxytocin in response to the sexual arousal induction showed larger decreases in oxytocin in response to mindful breathing. That is, perhaps engagement of oxytocin during tasks that elicit arousal (sexual arousal in our study, stress in Engert et al., 2016) are associated with more effective recovery processes (i.e., larger decreases in oxytocin during mindful breathing in our study, faster vagal recovery in Engert et al., 2016).

The current study has important implications for the treatment of sexual arousal concerns. Mindfulness-based therapies have shown promise in their effectiveness in treating female arousal difficulties. Our study demonstrated that women with greater arousability also showed greater neuroendocrine responses to both arousal and mindfulness inductions. This findings begs the question, could such neuroendocrine responsivity, specifically oxytocin responsivity, differentiate who may benefit the most from mindfulness-based interventions? Prior research has reflected the question presented above, demonstrating that mindfulness-based therapies are more effective for women with histories childhood sexual abuse (Brotto et al., 2012). Given that women with histories of childhood sexual abuse show different stress responses (Meston and Lorenz, 2013) and oxytocinergic profiles (Heim et al., 2008; Pierrehumbert et al., 2010), perhaps such modifications in the oxytocinergic system makes these women more sensitive to interventions that impact their oxytocin system, such as mindfulness training.

### Limitations and Future Directions

We argued that mindfulness leads to increased arousal, but our paradigm aimed to ensure assessment of sexual arousal that was not confounded from engaging in another task prior to the sexual arousal assessment. As such, all participants engaged in the sexual arousal task prior to engaging in the mindful breathing task. This paradigm also limited our ability to fully assess the process of recovery, which may have been particularly fruitful for understanding neuroendocrine correlates of mindfulness and sexual arousal. Future research would benefit from having some participants engage in an arousal task without engaging in mindful breathing (or another type of task), others engage in a mindful breathing task prior to the arousal task, as well as having other participants engage in mindful breathing task following the arousal task. Such a paradigm would allow for a more accurate understanding of *how* mindfulness practice is associated with augmentations in sexual arousal, as well as understanding the effect of mindfulness practice on recovery processes. Moreover, investigating how socially oriented forms of mindfulness practices differ from the more traditional focused breathing inductions may elucidate the complex pattern of results. Future research would benefit from larger sample sizes to obtain sufficient power to examine differences between women of various sexual orientation and interactions between the facets of trait mindfulness. Finally, inclusion of measures related to compassion and general social functioning will be important considerations for future research. Understanding how compassion and general social functioning are involved in oxytocin, mindfulness, and arousal would provide important insights into our results and could have important clinical implications.

The fact that our results established a relation between subjective and neuroendocrine responsiveness to mindful breathing and to sexual arousability stimulates questions regarding the potential mediators and causal pathways. An interesting line of inquiry would be to investigate the role of self-compassion in mindfulness, oxytocin, and sexual arousal. Among women with sexual interest and desire disorders, change in overall sexual function through mindfulness-based interventions was mediated by self-compassion, although mindfulness based treatment did not actually increase self-compassion during treatment (Paterson et al., 2017). Hence, future research may consider comparing the pathways by which mindfulness, compassion, and oxytocin enhance arousal (does oxytocin promotes changes in mindfulness through compassion? does compassion facilitate change in oxytocin that results from mindfulness? etc.). Such understanding holds great potential in identifying why some women benefit more or less from mindfulness-based interventions. Another potential mediator not investigated in the current study is vagus nerve activity (Carter, 2017). During sexual arousal, oxytocin conveys sensory activity from the cervix, preparing the genitals for orgasm via the vagus nerve (Komisaruk and Sansone, 2003). Innervation by vagus nerve also enhances emotion regulation, is involved in mindfulness, and may be linked to compassion (Carter, 2017; Carter et al., 2017). Moreover, future investigation into the causal pathways between oxytocin, mindfulness, and arousability would benefit from investigating the effects of exogenous (intranasal) administration on sexual arousal and mindfulness. The precise pathways by which oxytocin may promote and result from *both* mindfulness and sexual arousal is a critical next step for future research.

## Conclusion

In summary, the present research contributes to the growing number of studies indicating that mindfulness is associated with sexual arousal and provides an important first examination at the links between mindfulness, sexual arousal, and oxytocin. The current research contributed to our fundamental understanding of the role of attentional processes in sexual arousability, and the potential neuroendocrine underpinnings of these links. Specifically, results indicated that oxytocin plays a role in mindfulness, sexual arousal toward the preferred gender, and the link between the responsiveness to mindfulness and sexual arousability. Future research that continues to investigate both within-person and between-person variability in the neuroendocrine correlates of female sexual arousability will make important contributions to our evolving understanding of female sexuality.

## Ethics Statement

The study was approved by University of Utah Institutional Review Board.

## Author Contributions

LD had full access to all the data in the study and took responsibility for the integrity of the data collection. JD and LD took responsibility for the accuracy of the data analysis. JD conducted the study design and data analysis. All authors contributed to the write up in efforts consistent with author order.

## Conflict of Interest Statement

The authors declare that the research was conducted in the absence of any commercial or financial relationships that could be construed as a potential conflict of interest.
