# Corrigendum: Subjective and Oxytocinergic Responses to Mindfulness Are Associated With Subjective and Oxytocinergic Responses to Sexual Arousal

**DOI:** 10.3389/fpsyg.2019.01737

**Published:** 2019-10-18

**Authors:** Janna A. Dickenson, Jenna Alley, Lisa M. Diamond

**Affiliations:** ^1^Program in Human Sexuality, Department of Family Medicine and Community Health, University of Minnesota, Minneapolis, MN, United States; ^2^Diamond Laboratory, Department of Psychology, University of Utah, Salt Lake City, UT, United States

**Keywords:** oxytocin, sexual arousal, mindfulness, attentional shifts, women, sexual health

In the original article, there was an error. The citation for “Brotto et al., 2008” was incorrectly written. It should be “Brotto et al., 2012.”

A correction has been made to the **Discussion**, paragraph seven:

“The current study has important implications for the treatment of sexual arousal concerns. Mindfulness-based therapies have shown promise in their effectiveness in treating female arousal difficulties. Our study demonstrated that women with greater arousability also showed greater neuroendocrine responses to both arousal and mindfulness inductions. This findings begs the question, could such neuroendocrine responsivity, specifically oxytocin responsivity, differentiate who may benefit the most from mindfulness-based interventions? Prior research has reflected the question presented above, demonstrating that mindfulness-based therapies are more effective for women with histories childhood sexual abuse (Brotto et al., 2012). Given that women with histories of childhood sexual abuse show different stress responses (Meston and Lorenz, 2013) and oxytocinergic profiles (Heim et al., 2008; Pierrehumbert et al., 2010), perhaps such modifications in the oxytocinergic system makes these women more sensitive to interventions that impact their oxytocin system, such as mindfulness training.”

The reference has also been changed to reflect this correction from “Brotto, L. A., Basson, R., and Luria, M. (2008). A mindfulness-based group psychoeducational intervention targeting sexual arousal disorder in women. *J. Sex. Med*. 5, 1646–1659. doi: 10.1111/j.1743-6109.2008.00850.x” to “Brotto, L. A., Seal, B. N., and Rellini, A. (2012). Pilot study of a brief cognitive behavioral versus mindfulness-based intervention for women with sexual distress and a history of childhood sexual abuse. *J. Sex Marital Ther*. 38, 1–27. doi: 10.1080/0092623X.2011.569636”.

The authors apologize for this error and state that this does not change the scientific conclusions of the article in any way. The original article has been updated.
